# Investigation of olfactory receptor family 51 subfamily j member 1 (OR51J1) gene susceptibility as a potential breast cancer-associated biomarker

**DOI:** 10.1371/journal.pone.0246752

**Published:** 2021-02-10

**Authors:** Maryam Asadi, Nahid Ahmadi, Simin Ahmadvand, Ali Akbar Jafari, Akbar Safaei, Nasrollah Erfani, Amin Ramezani

**Affiliations:** 1 Shiraz Institute for Cancer Research, School of Medicine, Shiraz University of Medical Science, Shiraz, Iran; 2 Department of Molecular Medicine, School of Advanced Medical Sciences and Technologies, Shiraz University of Medical Sciences, Shiraz, Iran; 3 Department of Medical Biotechnology, School of Advanced Medical Sciences and Technologies, Shiraz University of Medical Sciences, Shiraz, Iran; 4 Department of Statistics, Yazd University, Yazd, Iran; 5 Department of Pathology, School of Medicine, Shiraz University of Medical Sciences, Shiraz, Iran; 6 Department of Immunology, School of Medicine, Shiraz University of Medical Sciences, Shiraz, Iran; Okayama University, JAPAN

## Abstract

Among cancer treatment methods, targeted therapy using cancer-associated biomarkers has minimum side effects. Recently olfactory receptor (OR) family attracts the researcher’s attention as a favorable biomarker of cancer. Here, a statistical approach using complete data from the human protein atlas database was used to evaluate the potential of OR51J1 gene as a cancer-associated biomarker. To confirm the findings of statistical analysis, the OR51J1 mRNA and protein expression levels in breast tumor and normal tissue were measured using quantitative Real Time PCR (qRT-PCR) and immunohistochemistry (IHC) techniques. The association with clinicopathological factors was analyzed. Statistical analysis revealed that OR51J1 has a high expression level in more than 20 types of cancer tissues without any expression in 44 normal tissues. In 15 cancer types, including breast cancer, expression score was more than 90%. The qRT-PCR analysis in breast cancer showed OR51J1 have significantly higher expression level in tumors than normal tissues (2.91 fold). The IHC results showed OR51J1 expression on other cellular subtypes than tumor and normal cells, including myoepithelium, fibroblast, and lymphocytes. OR51J1 protein expression in invasive cells, as well as its overall score, showed a significant correlation with ER and PR expression and breast cancer (BC) subtypes. Results revealed the potential of OR51J1 as a cancer-associated biomarker for the diagnosis of breast cancer at the mRNA level.

## Introduction

Breast cancer accounts for 25% of all women cancer cases and 12% of all cancer cases [1]. Since attacking the cancer tissue will also hurt normal cells, it is logical to target some specific markers that are expressed in most neoplastic cells but not in healthy tissues. [2]. Recently many types of cancer have been targeted to specifically inhibit cancer cells without any damage to normal tissues [3]. Cancer targeted therapies are drugs or other substances that react with cancer-specific molecules and block the growth of cancer and have shown much less toxicity than traditional chemotherapy [4]. An ideal tumor marker should be highly specific, highly sensitive, and also should be detected by simple cheap tests and easily obtainable specimens [5]. Most of the anticancer therapies in development are based on identifying targetable gene mutations and marker proteins that lead to more selective prevention methods, diagnosis, and treatment.

These tumor biomarkers may be the result of host response to the tumor [6], genetic mutations and/or the expression of new proteins in tumor cells [7]. They can be used for the early diagnosis, attacking cancer cells, following up the therapy, prognosis, and therapeutic response prediction [8]. Development in genomics, proteomics, and epitopes detection, elicits accurate immune responses and reduces the cost and time that is spent in the development process of targeted therapy [9].

In this study, OR51J1, a member of olfactory receptors, was selected for evaluation as a potential BC biomarker. At first, a statistical approach was used to evaluate the potential of OR51J1 gene as a cancer-associated biomarker. Confirming the findings of statistical analysis, the OR51J1 mRNA and protein expression levels in breast tumors and normal tissues were measured using qRT-PCR and IHC techniques and its association with clinicopathological factors was analyzed.

## Materials and methods

### *In silico* evaluation of OR51J1 as a potential tumor marker

To evaluate OR51J1 potential as a tumor marker, a statistical approach was used. Complete data from the human protein atlas database was downloaded as an XML file (https://www.proteinatlas.org/about/download, 2018). Several executable programs written in R 3.5.2 software [10] were provided to find the ideal genes with high expression levels in neoplastic tissues and low expression levels in normal tissues. Genes with no expression in the normal tissues but with a high expression level in the neoplastic tissues were selected from a panel of 13000 genes. Then using a scoring system, the selected proteins were sorted. The following formula was used for scoring: the highest score is the gene with the highest number of samples (at most 12 samples) with highest expression level (the expression level were ranked according to immunohistochemistry in four levels: high, medium, low and not detected) in most cancer type (in 21 different forms of human cancer) among the highest number of not detected samples in all normal tissues (about 45 normal human tissue types).

Scoring formula: $\frac{3A+2B+C+0D}{3\left(A+B+C+D\right)}\times 100$ Where *A*, *B*, and *C* are the number of samples with high, medium, and low expression levels, respectively, and *D* is the number of not detected samples.

Then, using another computable program, the number of cancers in which those proteins had expressed was determined, and those with the expression score higher than 50% were chosen. The mean score of each protein in different cancers was determined. Finally the qualitative data were transformed to quantitative data [11].

The expression level of OR51J1 gene was also evaluated in GEPIA (http://gepia.cancer-pku.cn/detail.php?gene=or51j1) cancer database using a similar procedure.

### OR51J1 mRNA expression quantification

#### Tissue samples preparation

Forty female breast cancer patients were selected based on their pathological reports. Tumor and adjacent normal tissue biopsies were removed by surgery, snap-freezing performed and stored at -70˚C. Those patients who received chemotherapy or radiotherapy were excluded. All data were fully anonymized before we accessed them. The samples were collected from 2015 to 2019 in MRI hospital, Shiraz, Iran. Patients signed written informed consent before surgery. The study was approved by the research ethics committee of Shiraz University of Medical Sciences (Approval ID: IR.SUMS.REC.1397.667). The project was found to be in accordance to the ethical principles and standard and national norms for conducting Medical Research in Iran. All tissues Clinico-pathological information was obtained from pathology reports.

#### RNA extraction and cDNA synthesis

Total RNA was extracted from snap-frozen tissues using RNX-Plus (CinnaGen, Iran) according to the manufacturer’s instruction. DNase treatment was performed using Fermentas DNase I (Fermentas, Lithuania) to eliminate genomic DNA. cDNA synthesis was performed with random hexamers and oligo-dT primers using First Strand cDNA Synthesis Kit (Fermentas, Lithuania).

#### Quantitative real-time PCR (qRT-PCR)

qRT-PCR was performed by ABI StepOne (Applied Biosystems, USA) in 48 well microtitre plates using a Sybergreen master mix (ABI, USA) and OR51J1 specific primers. The beta-actin gene was used as an internal control gene for data normalization. Amplification was performed under the following conditions: 95°C for 10 min for initial denaturation, 40 cycles of 95°C for 10 sec, and 60°C for 40 sec. Amplification efficiencies were calculated and included in data normalization. The specificity of the qRT-PCR was confirmed by melt curve analysis. Data normalization was performed using pfaffl formula [12,13]. The primer sequences for OR51J1 and beta actin genes were: OR51J1-F: CACTGGAGAAGGGAGGAAGAAG, OR51J1-R: GGAAGAAGAGGTAGGCATTGG, Bactin-F: GCCTTTGCCGATCCGC andBactin-R: GCCGTAGCCGTTGTCG.

### Immunohistochemical staining of breast tissue

#### Tissue samples preparation

A population of 69 IDC patients with available formalin-fixed and paraffin-embedded (FFPE) tissue blocks was retrospectively selected for OR51J1 expression analysis at the protein level. All the patients had received surgical removal of the primary tumor as their first-line treatment between 2009 and 2011 in Shahid Faghihi Hospital of Shiraz University of Medical Sciences, Shiraz, Iran. Patients’ data, including their clinicopathological characteristics and survival status were obtained from their records. all data were fully anonymized before we accessed them. Patients signed written informed consent before surgery. The study was approved by the Ethics Committee of Shiraz University of Medical Sciences.

#### Immunohistochemistry

FFPE tissue blocks were cut into 3μm sections and mounted on positively charged IHC slides. The sections were deparaffinized in 61°C for 15 minutes, immediately immersed in fresh xylene for 30 minutes, and rehydrated in decreasing graded ethanol solutions, each for 45 seconds. Epitopes were retrieved using heat-induced epitope retrieval method by Tris-EDTA, pH 9 for about 20 minutes. Endogenous peroxidases, as well as nonspecific hydrophobic protein-protein interactions, were blocked by the use of 10% H2O2 and 10% normal goat serum, respectively. Anti- OR51J1 antibody [Sigma, HPA017605, 1/300] were added to the sections and incubated for 45 minutes at room temperature and in a humid chamber. Visualization was performed according to the instruction of Master Diagnostic detection kit [MAD-000237QK]. Negative control was included by replacing primary antibody with PBS to check the specificity of secondary antibodies of the detection kit.

Finally, slides were dehydrated through graded ethanol solutions, counterstained with hematoxylin and permanently mounted in Entellan [Merck, 107961].

IHC-stained slides were assessed by an expert pathologist who was blinded to clinicopathologic characteristics of the patients. The expression of OR51J1 was evaluated in different cellular subtypes, including normal, *in situ*, and invasive ductal epithelial cells, fibroblasts, lymphocytes and endothelium. No expression was reported as negative, and in positive cells the results were reported as 1+, 2+ and 3+ for weak, moderate and strong expression, respectively. It should be mentioned that the IHC results for ER, PR and HER2 expression were obtained from patients’ pathology reports.

#### Statistical analysis

Results were analyzed using SPSS (version 16, SPSS Inc, USA) and GraphPad Prism 6 software (GraphPad Software, Inc., USA). Independent student T-test was conducted to explore the mean differences of OR51J1 mRNA expression among normal and tumor breast tissues as well as OR51J1 score differences among clinicopathological features of the study population. Only in case of Breast Cancer Subtypes we used one way ANOVA and LSD Post Hoc.

Scores for OR51J1 protein expression were obtained by Principal Component Analysis (https://bpspsychub.onlinelibrary.wiley.com/doi/epdf/10.1111/j.2044-8317.1970.tb00432.x). As this method omits cases with missing values, only the OR51J1 expression in invasive cells, lymphocytes and endothelial cells was considered for scoring to reduce the number of censored cases in the process of analysis. Pearson’s chi-squared (χ2) test or Fisher’s exact test were used to compare the association of high and low OR51J1 protein expression in invasive cell with categorical disease variables. *P*-values less than 0.05 were considered as significance level. In case of Fisher’s Exact Test and T-test, *P*-values of two-tailed test was considered”.

## Results

### *In silico* analysis

**Fig 1:**
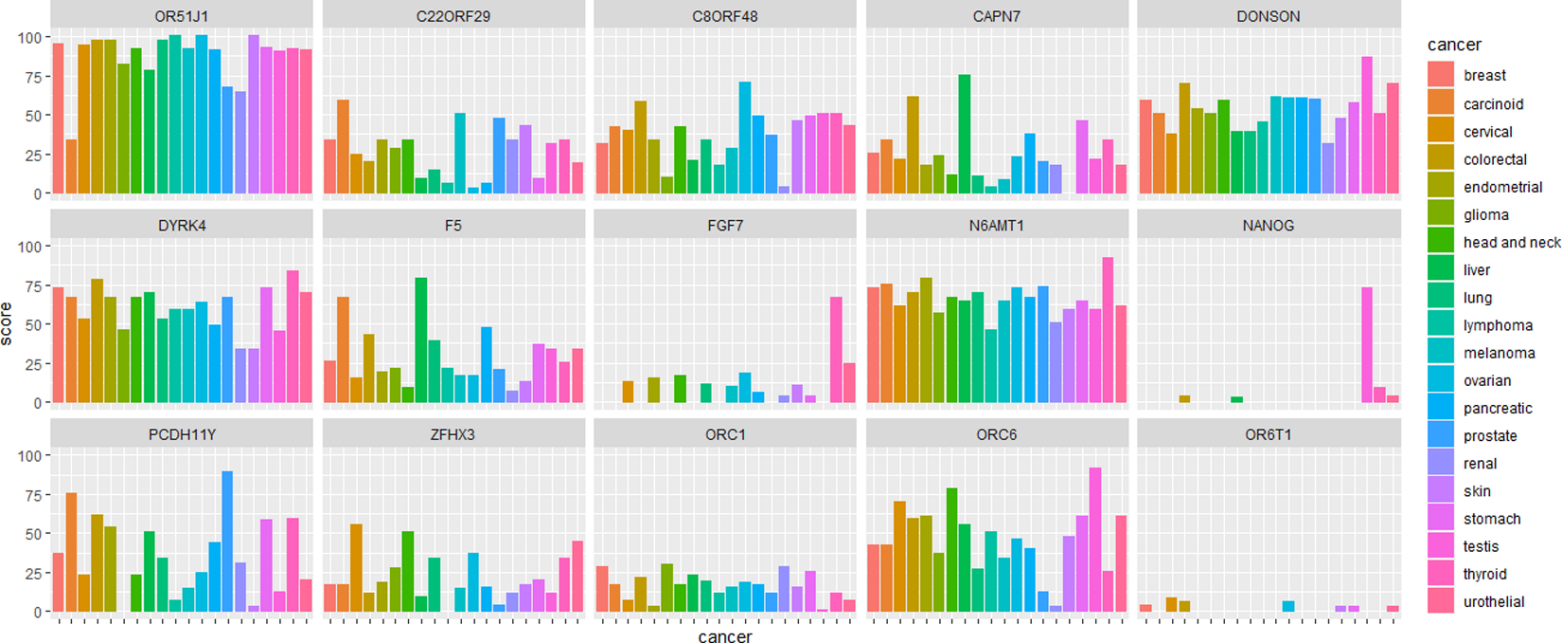
The protein expression score of OR51J1 and some of high score genes in different cancers. No expression was detected in normal tissues. Scoring formula: ((A*3) + (B*2) + (C) + (D*0))/((A+B+C+D)*3)*100 A, B, and C: The number of samples with high, medium and low expression levels, respectively. D: The number of not detected samples.

*In silico* analysis of human protein databases, revealed 56 genes didn’t express in normal tissues. Among them, the score of 12 genes was upper than 50% in at least one cancer. The OR51J1 gene achieved the highest ranking, and was expressed in 21 different types of malignancies. In 15 cancer types, its expression score was more than 90% (in Fig 1 some high score genes is showed). According to data of human protein atlas database there is no expression for OR51J1 in normal tissues. On the other hand according to GEPIA data base there is a level of expression for OR51J1in normal tissue but there are differences with expression in tumor tissues. And this difference in expression between tumor and related normal tissues is more seen in head and neck squamous cell carcinoma and ovarian serous cyst adeno carcinoma and breast cancers.

### OR51J1 mRNA expression quantification in breast cancer

**Fig 2:**
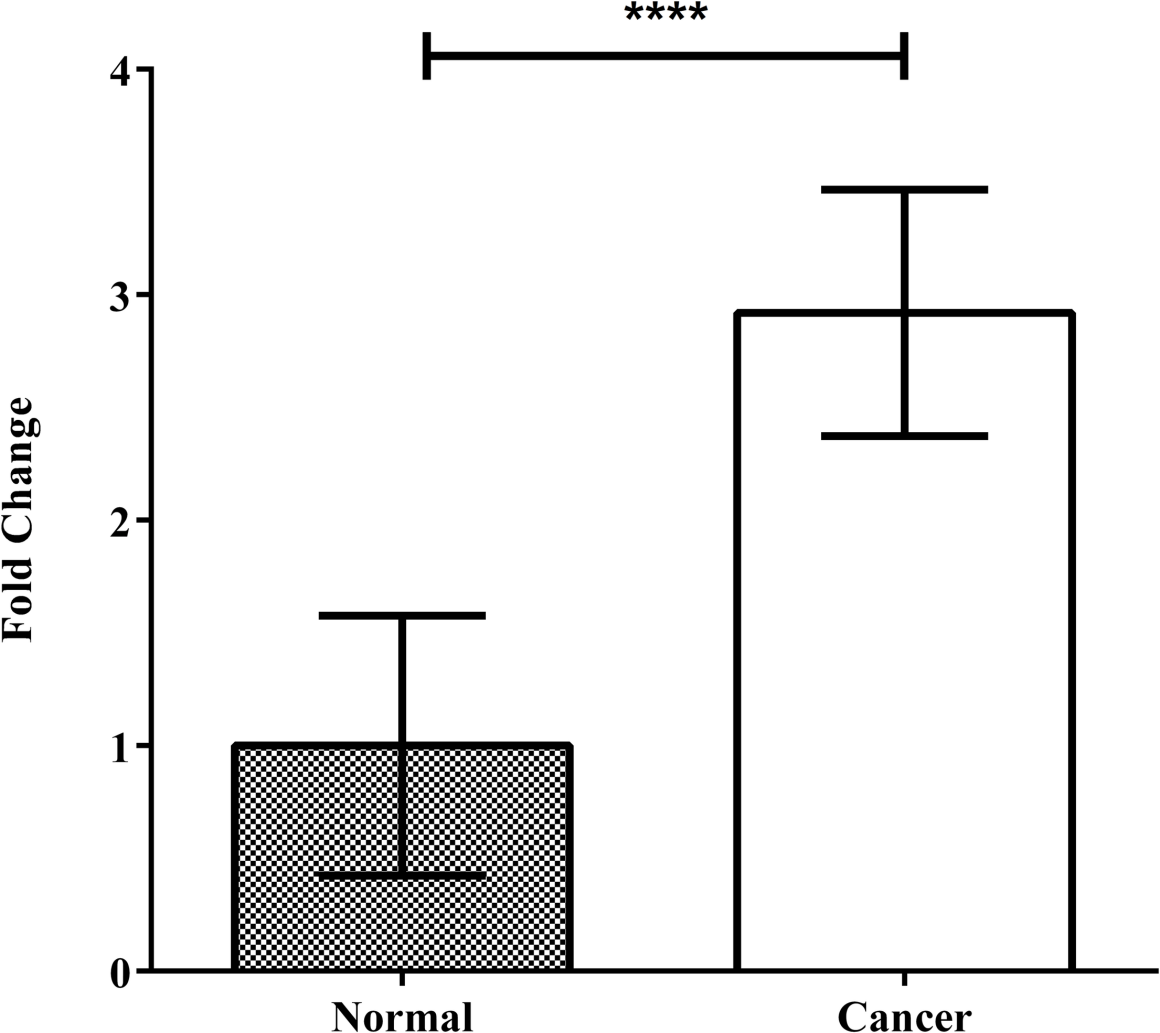
Comparison of relative gene expression of OR51J1 in tumor and adjacent normal tissues using qRT- PCR technique. Results were analyzed by the T test. *: P<0.05.

qRT-PCR analysis on 40 breast cancer patients revealed that OR51J1 mRNA had significantly higher expression levels in breast tumors than in adjacent normal tissues (*P*<0001). The quantitative expression pattern for OR51J1, (Fig 2) a comparison of the OR51J1 gene expression profile using unpaired t test showed a nearly 3-fold increase in breast tumors compared to normal tissues. As one of the common problems in RNA extraction is genomic DNA contamination, and to ensure that the PCR products were generated from cDNA and not genomic DNA, additional PCR reactions were carried out on extracted RNA samples without reverse transcription. No amplification was observed in these reactions.

Assessment of clinicopathological characteristics of breast cancer patients showed that the mean age was 50.9 ± 10.2 (31–79) years and 67% of patients were in early stages (I and II). The greatest dimension of tumor size among was ≤2 cm with tumor type in 35% of patients was invasive ductal carcinoma. More information about clinicopathological characteristics are shown in Table 1. Assessment of the relationship between the OR51J1 mRNA expression pattern with different breast cancer grade, stages, the number of involved LNs, tumor size and HER2, ER, and PR using one-way ANOVA revealed no significant relation (Table 1).

**Table 1:** Comparison of IDC breast cancer clinicopathological features based on the OR51J1 mRNA and protein expression and score level.

Variable		qRT-PCR	*P*-value^c^	OR51J1 expression*		*P*-value^d^	OR51J1 score**	*P*- value^c^
		Mean (SEM)^a^		Low (%)	High (%)		Mean (SEM)^a^	
**Age**	≤50	2.962515 (0.110071)	0.797	23 (33.3)	15 (21.7)	0.071	0.096153 (0.174188)	0.412
	>50	2.913182 (0.154564)		25 (36.2)	6 (8.7)		-0.112731 (0.183272)	
**Grade**	I+II	2.882622 (0.126062)	0.354	32 (47.1)	13 (19.1)	0.619	-0.090737 (0.157672)	0.228
	III	3.063223 (0.144155)		15 (22.1)	8 (11.9)		0.233634 (0.212351)	
**ER**	Positive	2.980943 (0.152694)	0.564	32 (46.4)	8 (11.6)	0.027	-0.259636 (0.158013)	0.009
	Negative	2.854519 (0.152398)		16 (23.2)	13 (18.8)		0.394646 (0.184570)	
**PR**	Positive	2.969573 (0.145303)	0.655	27 (39.1)	5 (7.2)	0.013	-0.365161 (0.173832)	0.005
	Negative	2.868944 (0.175185)		21 (30.4)	16 (23.2)		0.331965 (0.163050)	
**HER2**	Positive	3.013533 (0.099298)	0.281	12 (17.4)	10 (14.5)	0.064	0.236394 (0.192528)	0.164
	Negative	2.731462 (0.338261)		36 (52.2)	11 (15.9)		-0.118197 (0.161064)	
**Breast cancer sub-type**	Luminal	2.980943 (0.152694)^b^	0.834	32 (46.4)	8 (11.6)	0.059^e^	-0.259636 (0.158013)^b^	0.036
	HER2 enriched	2.916357 (0.082827)		6 (8.7)	7 (10.1)		0.365094 (0.260598)	
	TNBC	2.751457 (0.430586)		10 (14.5)	6 (8.7)		0.426661 (0.272778)	
**LN status**	Positive	2.805733 (0.205747)	0.246	21 (31.8)	11 (16.7)	0.485	0.130534 (0.167649)	0.267
	Negative	3.031802 (0.080058)		25 (37.9)	9 (13.6)		-0.161204 (0.198709)	
**TNM stage**	I+II	2.951461 (0.098637)	0.887	38 (55.1)	16 (23.2)	0.761^e^	0.006061 (0.141417)	0.933
	III	2.908084 (0.389288)		10 (14.5)	5 (7.2)		-0.021214 (0.286650)	

ER: Estrogen Receptor; PR: Progesterone Receptor; HER2: Human Epidermal Growth Factor Receptor 2.

*OR51J1 expression on invasive cells only; Low: 1+/2+; High: 3+.

**OR51J1 score based on the OR expression by invasive cells, lymphocytes and endothelial cells. Principal factor Analysis method was used for scoring.

a: Independent student T-test;

b: One-way ANOVA;

c: *P*-values of two-tailed Test.

d: *P*-values of Chi-square test;

e: *P*-values of Fisher’s exact test (two-tailed).

### OR51J1 protein expression evaluation

None of the 69 female patients with IDC breast cancer had received chemotherapy, radiotherapy, or neoadjuvant therapy before their surgery. The mean age of patients at the diagnosis was 49.32 (25 to 81) years. The mean of follow up period was 1862.58 (248 to 2705) days. According to 7^th^ edition of AJCC TNM classification system [14] 44 (63.8%) patients of our study population belonged to stage II and none of them was stage IV at the time of diagnosis (Table 1).

#### Association of OR51J1 protein expression on invasive ductal cells with disease parameters

**Fig 3:**
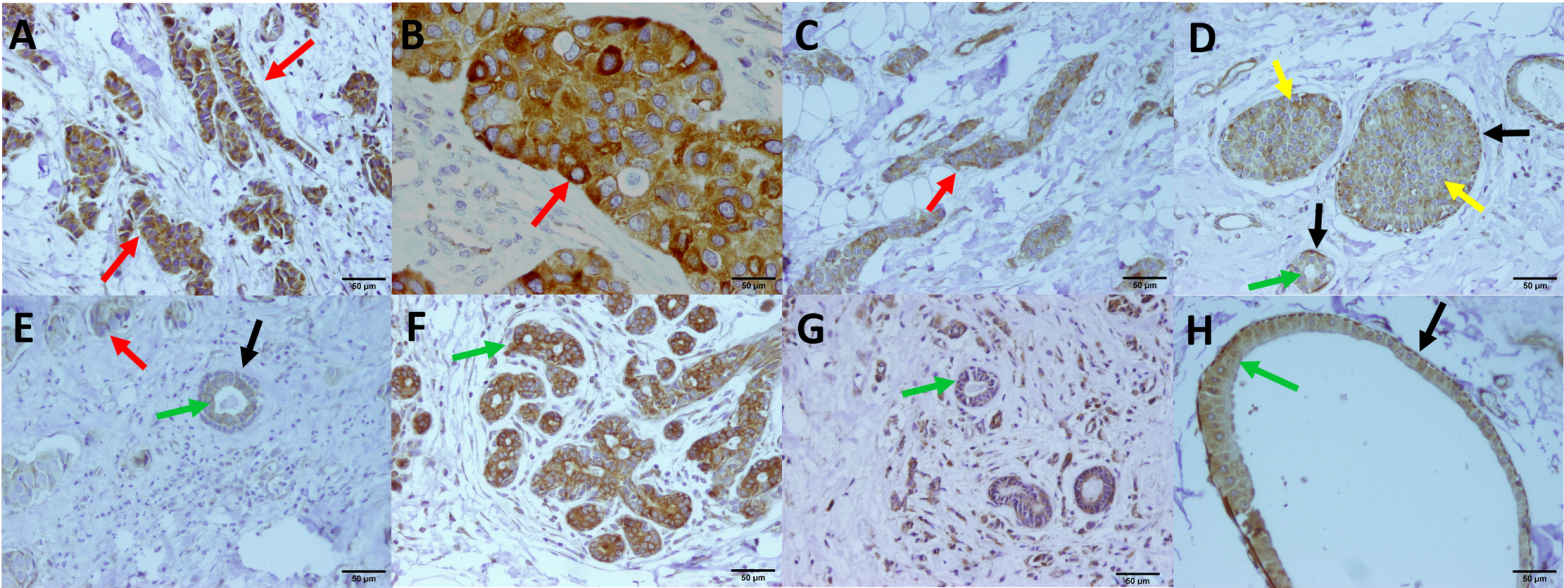
Representative images of OR51J1 expression in different ductal cells. Red arrows show invasive cells with high (A and B), moderate (C) and low (E) protein expression. Yellow arrows show *in situ* cells with moderate (D) expression. Green arrows illustrate normal ducts with high (F), intermediate (D) and low (E) expression. The black arrows show myoepithelial cells with high (C and H) and negative (E) OR51J1 protein expression (C). *Photomicrographs were taken at 200 magnifications.

According to the IHC results, OR51J1 was expressed in different cellular subtypes including normal, *in situ* and invasive ductal epithelial cells as well as, myoepithelium, fibroblasts, tumor infiltrating lymphocytes and endothelium (Fig 3). However, to study the association of OR51J1 with clinicopathological characteristics of the disease, protein expression by invasive cells was selected. In this regard, invasive cells were first dichotomized into low (1+ and 2+) (n = 48, 69.6%) and high (3+) (n = 21, 30.4%) groups based on OR51J1 protein level, and then compared with disease parameters (Table 1). Among different features, the low OR51J1 expression level was associated with ER (*P* = 0.027) and PR positivity (*P* = 0.013). No significant associations were observed between OR51J1 expression and histological grade (P = 0.619), lymph node involvement status (P = 0.485) and TNM-stage (P = 0.761). Breast cancer subtypes were defined as luminal (ER+ and/or PR+), HER2-enriched (ER-, PR-, HER2+), and triple negative (TNBC) (ER-, PR-, HER-) and their association with OR51J1 expression were evaluated and no significant correlation was observed (P = 0.059).

#### Association of OR51J1 score with disease parameters

**Fig 4:**
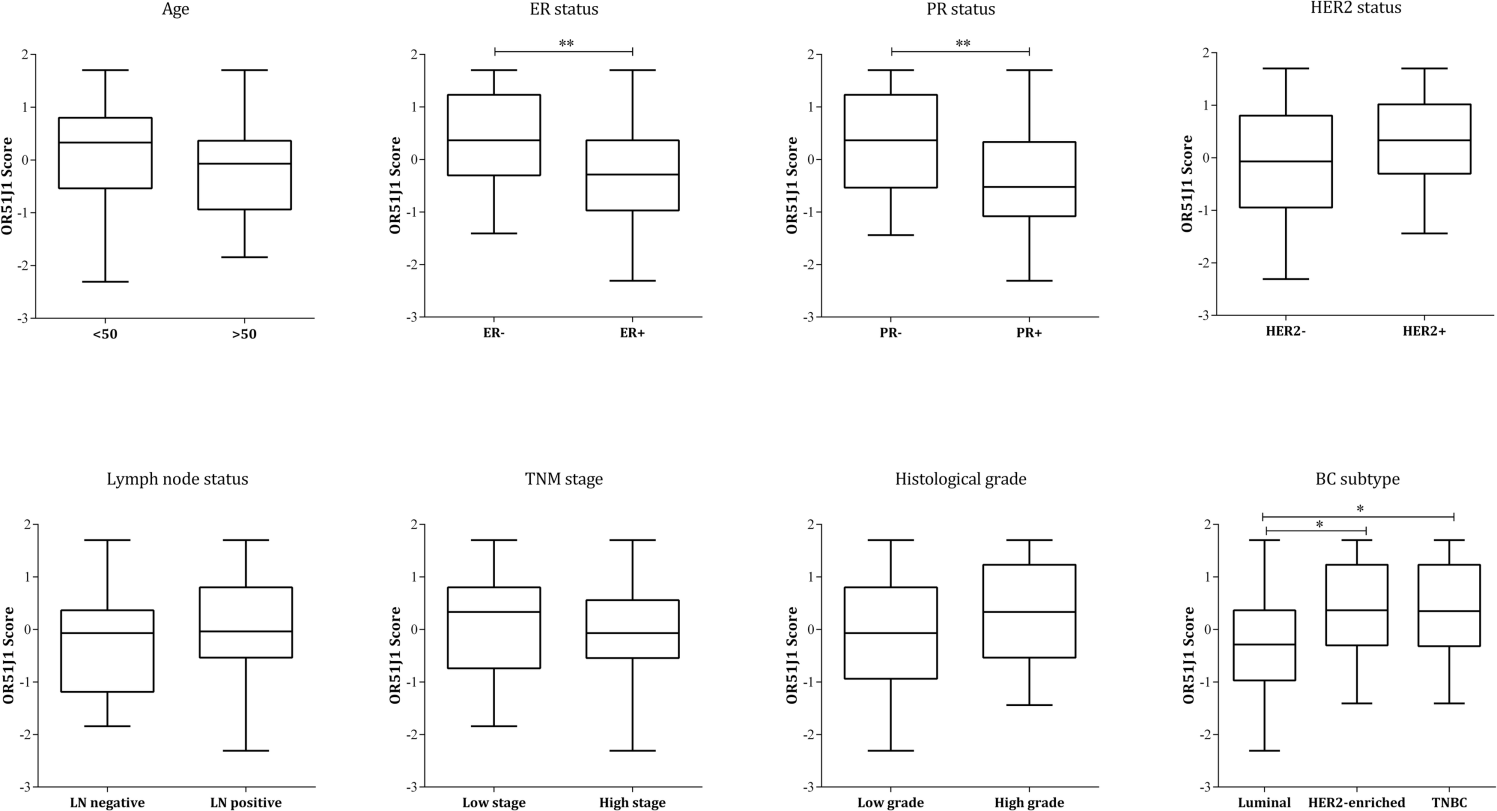
Comparison of OR51J1 scores level among breast cancer parameters. Scores were obtained through the principal factor scoring method. As it is interpreted from the graphs, scores are significantly lower in ER+, PR+ tissues than ER- and PR- ones. There are also significant differences between the scores of luminal breast cancers with both HER2-enriched and TNBC subtypes.* P≤0.05 has been considered as significant level.

An informative score that considers the expression status of OR51J1 protein in different cellular subtypes of every single patient was created for further analysis of the whole effect of OR51J1 protein expression on disease parameters. As the principal factor scoring method omits cases with missing values, only the OR51J1 expression on invasive cells, lymphocytes and endothelial cells was considered to reduce the number of censored cases in the process of scoring. Comparing the mean of scores among different clinicopathological features showed a significant difference between ER- and ER+ (*P* = 0.009) as well as PR- and PR+ (*P* = 0.005) groups. The one way ANOVA also indicated significant difference among BC subtypes (*P* = 0.036). LSD Post Hoc showed that the mean of OR51J1 score in luminal BCs was significantly lower than the HER2-enriched (P = 0.048) and TNBC (P = 0.035), respectively (Fig 4).

## Discussion

Among cancer treatment methods, targeted therapy using cancer-associated biomarkers has the least side effects. To achieve more predictive, preventive, diagnostic, and therapeutic methods, anticancer therapies have been developed based on the recognition of tumor markers and targetable gene mutations [2]. In this study high throughput data were minded to evaluate a potential new cancer-associated biomarker by comparing the expression data in normal and cancer tissues. OR51J1, a seven transmembrane protein of olfactory receptors (ORs), gained the highest ranking with the highest score. Olfactory receptors (OR) or odorant receptors are the members of G-protein coupled receptors (GPCRs) family. Their expression in non- olfactory tissues demonstrates their role in various cellular processes such as tissue injury, repair and regeneration promotion [15], cancer cell invasiveness promotion, metastasis emergence [16], and chemotaxis in sperm [17]. Some evidence mentioned ORs role in several diseases, including cancer [18]. Recently OR family has attracted the researcher’s attention as a favorable biomarker of cancer. Lea Weber et al. by immunohistochemical staining of OR2B6 in breast carcinoma tissues, revealed a distinct staining pattern of carcinoma cells from normal cells [19]. Researchers from Bochum detected OR10H1 in the human urinary bladder with notably higher expression at mRNA and protein levels in bladder cancer tissues [20]. Another study reported a high expression level of OR51B4 in the colon cancer cell line, HCT116, and in native human colon cancer tissues. Their results showed receptor activation leads to inhibition of cell proliferation and apoptosis [21]. In hepatocellular carcinoma, OR1A2, which is activated by monoterpene–Citronellol, reduced cell proliferation [22]. The activation of OR51E2 in prostate cancer, by its ligand b-Ionon, leads to decreased cell proliferation and migration [23]. The same effect was observed in the OR2J3 receptor, which reduced cell proliferation after stimulation with Helional in human airway smooth muscle cells [24].

This study evaluated OR51J1 mRNA expression level in breast cancer and found the 2.9 fold higher expression in tumors compared to adjacent normal tissues and a low expression level of OR51J1gene was observed in normal tissues. As can be observed the results of qRT-PCR regarding the expression of OR51J1 in normal tissues are not consistent with statistical analysis. This result is supported by some evidence that mentioned the role of other OR family members (OR2B6, OR10H1, and OR51B4) in several diseases including cancer [19–21].

This study evaluated OR51J1 mRNA expression level in breast cancer and found the 2.9 fold higher expression in tumors compared to adjacent normal tissues and a low expression level of OR51J1gene was observed in normal tissues. As can be observed the results of qRT-PCR regarding the expression of OR51J1 in normal tissues are not consistent with statistical analysis. This result is supported by some evidence that mentioned the role of other OR family members (OR2B6, OR10H1, and OR51B4) in several diseases including cancer [19–21].

The study of OR51J1 protein expression in FFPE tissue sections of 69 IDC patients revealed the expression of this protein by invasive cells as well as other cellular subtypes of IDC tissues. Due to the expression of OR51J1 protein by the invasive cells in all samples and because of the clinical importance of these neoplastic cells, we first compared disease parameters based on the level of OR51J1 expression in invasive cells. According to the results, expression of this protein showed significant association with inherent features of cancer cells, including ER, PR and HER2 expression status, and no relation was observed with parameters like histological grade and TNM stage. Since OR51J1 protein also expressed in other cell types of the samples, for each patient a score was generated that was representative of the protein expression status in invasive cells, tumor infiltrating lymphocytes and endothelial cells. Based on the analyses, this score was also associated with intrinsic features of cancer cells including ER and PR expression status and BC subtype. These relations were not meaningful at the RNA expression level. As shown in IHC staining, IDC tumor samples potentially contain different OR51J1 positive cell types. As, through RNA preparation process, mRNA will be extracted from all of these cellular subtypes, it may be the underlying cause of different results of qRT-PCR and IHC. On the other hand, it should be noted that the low expression level of the genes at the mRNA level can be detected using the qRT-PCR technique, while the sensitivity of the IHC is not so great. In addition, post-translational modifications of proteins and the mRNA half-life can be another factors for the observed difference in the expression level of mRNA and protein.

Expression of ER, PR and HER2 have a profound effect on breast cancer outcome because of (at least) following reasons: a) signaling through these receptors drive cancer cell growth and proliferation, b) they are key biomarkers to define patient’s BC subtypes and guide the physicians to choose the best treatment regimen. In the current study, we observed that both low expressions of OR51J1 and its lower scores were associated with positive hormone receptor expressions. On the contrary, OR51J1 low expression had a negative effect on HER2 expression, although the results were statistically non-significant. Additionally, it has been reported that other members of OR family also have effects on cell proliferation and migration in several healthy and pathologic conditions [25]. Although this is the first study on OR51J1, studies on OR51E2, another member of this family, which is expressed in melanoma [26] and prostate cancer [27] revealed that its activation via its ligand, β-ionone, inhibits cancer cell proliferation. Another member, OR51B4, is also over-expressed in colorectal cancer and induces apoptosis and limits cell migration and proliferation through activation by its ligand, Troenan. All of these signify the importance of supplementary studies to find the probable signaling pathways by which ORs control cell proliferation. In the case of OR51J1, we suggest investigating further the possible molecular mechanisms by which OR51J1 affect ER, PR and HER2 expression and vice versa.

Finally, due to the valuable results of OR51J1 score, repeating this study with larger sample size, finding a proper cut-off point, and studying its power in separating patients with good and poor prognosis seems to be valuable. Furthermore, statistical analysis and qRT-PCR results revealed the potential of OR51J1 as a cancer-associated biomarker for the diagnosis of breast cancer at the mRNA level. But further testing is needed to explicitly introduce this gene as a biomarker. At least with the IHC cannot be introducing OR51J1 as a biomarker firmly.

## Supporting information

S1 File (RAR)

S2 File (RAR)

S3 File (SAV)

S4 File (SAV)
